# Robust Control for the Segway with Unknown Control Coefficient and Model Uncertainties

**DOI:** 10.3390/s16071000

**Published:** 2016-06-29

**Authors:** Byung Woo Kim, Bong Seok Park

**Affiliations:** 1 Department of Electronic Engineering, Chosun University, 375 Seosuk-Dong, Dong-Gu, Gwangju 61452, Korea; oocsos@naver.com; 2 Division of Electrical, Electronic, and Control Engineering, Kongju National University, 1223-24 Cheonan-Daero, Seobuk-Gu, Cheonan 31080, Korea

**Keywords:** unknown control coefficient, Segway, prescribed performance function, Nussbaum gain technique, model uncertainty

## Abstract

The Segway, which is a popular vehicle nowadays, is an uncertain nonlinear system and has an unknown time-varying control coefficient. Thus, we should consider the unknown time-varying control coefficient and model uncertainties to design the controller. Motivated by this observation, we propose a robust control for the Segway with unknown control coefficient and model uncertainties. To deal with the time-varying unknown control coefficient, we employ the Nussbaum gain technique. We introduce an auxiliary variable to solve the underactuated problem. Due to the prescribed performance control technique, the proposed controller does not require the adaptive technique, neural network, and fuzzy logic to compensate the uncertainties. Therefore, it can be simple. From the Lyapunov stability theory, we prove that all signals in the closed-loop system are bounded. Finally, we provide the simulation results to demonstrate the effectiveness of the proposed control scheme.

## 1. Introduction

The Segway is a vehicle extended from the inverted-pendulum system and balancing robot. It can go anywhere and is easy to manipulate. Thus, the Segway is becoming more prevalent on urban sidewalks and the stable controller is essential for human safety. In order to design the controller for the Segway, the linear controllers such as proportional-integral-derivative (PID) [1] and linear quadratic regulator (LQR) [2] were firstly proposed. The structure of these linear controllers is simple and it is easy to analyze the stability. However, they require the linearized model of the Segway to design the controller. This implies that there is a limit due to the narrow operating range. To solve this problem, various nonlinear control methods such as sliding mode control [3,4] and adaptive control [5,6] based on the backstepping technique [7] were proposed. It is well known that the backstepping technique requires the differentiation of the virtual control and this complicates the controller. Although the dynamic surface control method [8] can remove the disadvantage of the backstepping technique, it is still complex because it should use the adaptive technique [9,10], neural network [11,12,13], and fuzzy logic [14,15] to deal with the uncertainties.

To reduce the complexity of the nonlinear control methods, a low complexity control method was recently proposed [16]. By using the prescribed performance function, it can adjust the transient and steady-state responses. Further, it does not require the adaptive technique, neural network, and fuzzy logic to compensate the uncertainties. Hence, the controller can be implemented more simply. In this regard, several controllers for various applications were presented using this method. In [17], the adaptive dynamic surface control for nonlinear time-varying system was proposed. The output feedback controller for interconnected time-delay systems was presented in [18]. The robust formation controller for nonlinear multi-agent systems was proposed in [19]. However, all these works assume that the control coefficient is known or constant if it is unknown. This assumption is not applicable to the Segway because the control coefficient is time-varying and unknown. Therefore, we need to relax this assumption. Furthermore, the Segway is an underactuated system which has only one control input. Thus, it is difficult to design the controller because we should control the angle and velocity of the Segway, simultaneously.

Motivated by these observations, we propose a robust control method for the Segway in the presence of the unknown control coefficient and model uncertainties. Firstly, we employ the Nussbaum gain technique [20] to deal with the unknown time-varying control coefficient. Then, the robust controller using the prescribed performance function and the auxiliary variable is designed to compensate the uncertainties and solve the underactuated problem. For the stability of the proposed scheme, we prove that all error signals of the closed-loop control system are bounded using the Lyapunov stability theory. Finally, the simulation results are provided to demonstrate the effectiveness of the proposed control method. Compared with previous methods for the Segway, the main contribution of this paper is as follows: (i) The proposed approach can provide the desired performance of the tracking error without knowing the time-varying control coefficient; (ii) adaptive technique, neural network, and fuzzy logic, which make the controller complex, are not required to compensate the uncertainties and thus, the proposed scheme can be simple; (iii) by introducing an auxiliary variable, we can solve the underactuated problem.

The rest of this paper is organized as follows. The problem formulation is introduced in Section 2. In Section 3, the approximation-free control for the Segway is presented. In Section 4, the effectiveness of the proposed scheme is validated through simulation results. Finally, we conclude the paper in Section 5.

## 2. Problem Formulation

**Figure 1:**
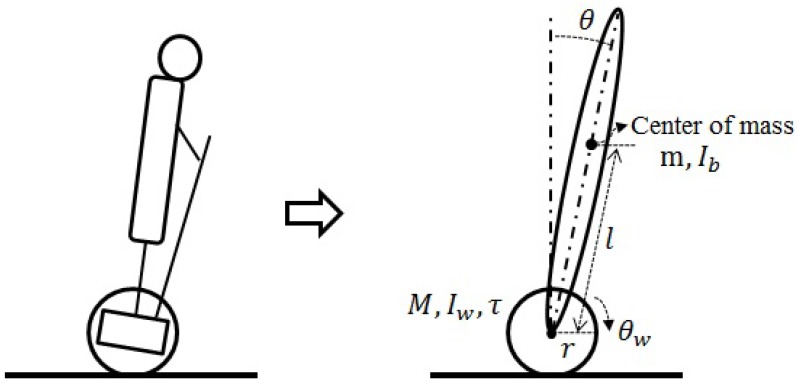
Segway model [22].

Consider the Segway model shown in Figure 1. The dynamics of the Segway is as follows [21].

(1) $$\begin{array}{c} m_{11}{\ddot{\theta }}_{w}+m_{12}\ddot{\theta }\cos\theta =\tau +m_{12}{\dot{\theta }}^{2}\sin\theta \\ m_{12}{\ddot{\theta }}_{w}\cos\theta +m_{22}\ddot{\theta }=-\tau +G_{b}\sin\theta \end{array}$$

where

$$\begin{array}{cc} & m_{11}=\left(m+M\right)r^{2}+I_{w}\\ & m_{12}=mlr\\ & m_{22}=ml^{2}+I_{b}\\ & G_{b}=mgl \end{array}$$

here, *m* is the mass of the body that is composed of the Segway base and the passenger, *M* is the mass of the wheel, *l* is the length between the wheel axle and the center of gravity of the body, $\theta_{w}$ and *θ* are wheel’s rotation angle and the inclination angle of the body, respectively, $I_{w}$ and $I_{b}$ are the moments of inertia of the body and the wheel, respectively, *r* is the radius of the wheel, and *τ* is the control torque applied to the wheels of the Segway.

From Equation Equation (1), it follows that

(2) $$\begin{array}{c} M_{1}{\dot{\theta }}_{w}+M_{2}\dot{\theta }=G_{b}\sin\theta +m_{12}{\dot{\theta }}^{2}\sin\theta \end{array}$$

where

$$\begin{array}{cc} & M_{1}=m_{11}+m_{12}\cos\theta \\ & M_{2}=m_{22}+m_{12}\cos\theta \end{array}$$

To make the state model of the Segway, we define the state variable as $x_{1}=\theta$ and $x_{2}=\dot{\theta }$. From Equation (2), we can represent Equation (1) as follows:

(3) $$\begin{array}{c} {\dot{x}}_{1}=x_{2}\\ {\dot{x}}_{2}=f\left(x_{1},x_{2}\right)-b\left(x_{1}\right)\tau \end{array}$$

where

$$\begin{array}{cc} & f\left(x_{1},x_{2}\right)=\{\left(m_{12}-{\left(m_{12}x_{2}\right)}^{2}\cos x_{1}\sin x_{1}+m_{11}G_{b}\sin x_{1}\right\}/\bar{M}\left(x_{1}\right)\\ & b\left(x_{1}\right)=M_{1}\left(x_{1}\right)/\bar{M}\left(x_{1}\right)\\ & \bar{M}\left(x_{1}\right)=m_{11}m_{22}-{\left(m_{12}\cos x_{1}\right)}^{2} \end{array}$$

In Equation (3), the velocity model of the Segway is omitted. This is because the Segway is underactuated. However, it is necessary to control the angular velocity of the wheel as well as the inclination angle. It will be solved by introducing an auxiliary variable.

**Assumption 1.**  The angle $x_{1}$ satisfies $-\pi /2<x_{1}<\pi /2$.

**Assumption 2.**  The state variables $x_{1}$, $x_{2}$, and ${\dot{\theta }}_{w}$ are measurable exactly by sensors such as accelerometer and gyroscope [23,24].

**Remark 1.**  In practice the sensor noise is inevitable. Thus, various techniques such as the Kalman filter [25] and state estimation [26] are used to reduce the effect of the sensor noise. However, the related technique for noise is another problem in view of the controller design. Therefore, we design the controller under Assumption 2.

In Equation (3), we assume $f(x_{1},x_{2})$ and $b(x_{1})$ are unknown. Further, $b(x_{1})$ is time-varying. Therefore, $f(x_{1},x_{2})$ and $b(x_{1})$ denote model uncertainties and unknown time-varying control coefficient, respectively. The *control objective* is to design the controller so that $x_{1}$ tracks its desired value $x_{d}=0^{\circ }$ while the control errors remain within the prescribed performance bounds even though there exist the unknown time-varying control coefficient and model uncertainties.

## 3. Controller Design

In this section, an approximation-free controller is designed step by step for the Segway with unknown time-varying control coefficient and model uncertainties. Define the errors as

(4) $$\begin{array}{c} \epsilon_{1}=\ln\frac{1+z_{1}}{1-z_{1}},\;\epsilon_{2}=\ln\frac{1+z_{2}}{1-z_{2}} \end{array}$$

where

$$\begin{array}{c} z_{1}=\frac{x_{1}}{\rho_{1}},\;z_{2}=\frac{x_{2}-\alpha -\mu }{\rho_{2}} \end{array}$$

here, *α* is a virtual control, *μ* is an auxiliary variable, and $\rho_{1}$ and $\rho_{2}$ are performance functions defined by

(5) $$\begin{array}{c} \rho_{1}\left(t\right)=\left(\rho_{1}\left(0\right)-\rho_{1}\left(\infty \right)\right)e^{-l_{1}t}+\rho_{1}\left(\infty \right)\\ \rho_{2}\left(t\right)=\left(\rho_{2}\left(0\right)-\rho_{2}\left(\infty \right)\right)e^{-l_{2}t}+\rho_{2}\left(\infty \right) \end{array}$$

where $\rho_{1}\left(0\right)>\left|x_{1}\left(0\right)\right|$ and $\rho_{2}\left(0\right)>\left|x_{2}\left(0\right)-\alpha \left(0\right)\right|$ are initial values of *ρ*-functions, $l_{1}$ and $l_{2}$ are gains of *ρ*-functions, $\rho_{1}\left(\infty \right)$ and $\rho_{2}\left(\infty \right)$ are final values of *ρ*-functions, α(0) is the initial value of the virtual control input *α*. In Equation (4), $z_{i}=\tanh\left(\epsilon_{i}/2\right)$ where i=1,2. Thus, if $\epsilon_{i}$ is bounded, $z_{i}$ satisfies $|z_{i}|<1$. This means that the tracking error is bounded such that $-\rho_{1}<x_{1}<\rho_{1}$.

**Remark 2.**  *As stated, it is difficult to control the inclination angle θ of the body and angular velocity ${\dot{\theta }}_{w}$ of the wheel simultaneously because there is only one control torque. However, we need to control the angular velocity of the wheel as well as the inclination angle of the body. To solve this problem, we introduce an auxiliary variable μ satisfying the differential equation*

(6) $$\begin{array}{c} \dot{\mu }=-k_{\mu }\mu +\gamma_{1}\tanh\left({\dot{\theta }}_{w}\right) \end{array}$$

*where $k_{\mu }$ and $\gamma_{1}$ are positive constants. From Equation (6), one can easily show that the auxiliary variable μ is bounded.*

Using Equations (3), (4) and (6), the error dynamics of $\epsilon_{1}$ and $\epsilon_{2}$ can be written as

(7) $$\begin{array}{c} {\dot{\epsilon }}_{1}=\frac{2{\dot{z}}_{1}}{1-z_{1}^{2}}=2\cosh^{2}\left(\epsilon_{1}/2\right)\frac{\alpha +\mu +\tanh\left(\epsilon_{1}/2\right)\rho_{2}-\tanh\left(\epsilon_{1}/2\right){\dot{\rho }}_{1}}{\rho_{1}}\\ {\dot{\epsilon }}_{2}=\frac{2{\dot{z}}_{2}}{1-z_{2}^{2}}=2\cosh^{2}\left(\epsilon_{2}/2\right)\frac{f\left(x_{1},x_{2}\right)-b\left(x_{1}\right)\tau -\dot{\alpha }+k_{\mu }\mu -\gamma_{1}\tanh\left({\dot{\theta }}_{w}\right)-\tanh\left(\epsilon_{2}/2\right){\dot{\rho }}_{2}}{\rho_{2}} \end{array}$$

To deal with the unknown time-varying control coefficient $b(x_{1})$, we employ the Nussbaum gain technique [20]. A function N(ζ) is called a Nussbaum function if it has the following properties.

$$\begin{array}{c} \lim_{s\to \infty }\sup\int_{s_{0}}^{s}N\left(\zeta \right)d\zeta =+\infty \\ \lim_{s\to \infty }\inf\int_{s_{0}}^{s}N\left(\zeta \right)d\zeta =-\infty \end{array}$$

In this paper, the Nussbaum function N(ζ)=cosh(ζ)sin(ζ) is considered and the following lemma is used to analyze the stability.

**Lemma 1.**  *Let V(·) and ζ(·) be smooth functions defined on $\left[0,t_{f}\right)$ with V(t)≥0, $\forall t\in [0,t_{f})$. For $t\in [0,t_{f})$, if the following inequality holds [27]:*

(8) $$V\left(t\right)\leq c_{0}+e^{-c_{1}t}\int_{0}^{t}bN\left(\zeta \right)\dot{\zeta }e^{c_{1}\varrho }d\varrho +e^{-c_{1}t}\int_{0}^{t}\dot{\zeta }e^{c_{1}\varrho }d\varrho$$

*where $c_{0}$ and $c_{1}$ are bounded constants, and b is unknown time-varying control coefficient, then V(t), ζ and $\int_{0}^{t}bN\left(\zeta \right)\dot{\zeta }d\varrho$ are bounded on $\left[0,t_{f}\right)$. According to [28], if the solution of the resulting closed-loop is bounded, then $t_{f}=\infty$.*

**Proof of Lemma 1.**  See Theorem 1 in [27]. ☐

**Remark 3.**  Lemma 1 means that if the condition Equation (8) is satisfied, the tracking error of the closed-loop system is bounded on [0,t). Furthermore, it can be extended for t=∞. Therefore, we will design the controller to satisfy the condition Equation (8).

Now the controller is designed step by step using the backstepping technique. Note that the backstepping technique has the disadvantage that requires the differentiation of the virtual control. However, the prescribed performance function based controller does not require the differentiation of the virtual control and thus, we can reduce the complexity of the controller.

*Step 1*: Consider the following Lyapunov function candidate for $\epsilon_{1}$

(9) $$\begin{array}{c} V_{1}=\frac{1}{2}\epsilon_{1}^{2} \end{array}$$

The time derivative of Equation (9) along with Equation (7) is

(10) $$\begin{array}{c} {\dot{V}}_{1}=\frac{\delta_{1}}{\rho_{1}}\epsilon_{1}\left(\alpha +\mu +\tanh\left(\epsilon_{2}/2\right)\rho_{2}-\tanh\left(\epsilon_{1}/2\right){\dot{\rho }}_{1}\right) \end{array}$$

where $\delta_{1}=2\cosh^{2}\left(\epsilon_{1}/2\right)>0$. The virtual control law *α* is chosen as

(11) $$\begin{array}{c} \alpha =-k_{1}\epsilon_{1}-\mu \end{array}$$

where $k_{1}$ is a positive constant. Substituting Equation (11) into Equation (10) yields

(12) $$\begin{array}{c} {\dot{V}}_{1}=\frac{\delta_{1}}{\rho_{1}}\epsilon_{1}\left(-k_{1}\epsilon_{1}+\tanh\left(\epsilon_{2}/2\right)\rho_{2}-\tanh\left(\epsilon_{1}/2\right){\dot{\rho }}_{1}\right) \end{array}$$

By the definition of Equation (5), $\rho_{2}$ and ${\dot{\rho }}_{1}$ are bounded. This means that there exists a positive constant $\Phi_{1}$ such that $|\tanh\left(\epsilon_{2}/2\right)\rho_{2}-\tanh\left(\epsilon_{1}/2\right){\dot{\rho }}_{1}|\leq \Phi_{1}$. Thus Equation (12) can be rewritten as

(13) $$\begin{array}{c} {\dot{V}}_{1}\leq \frac{\delta_{1}}{\rho_{1}}(-k_{1}|\epsilon_{1}{|}^{2}+\Phi_{1}|\epsilon_{1}\left|\right) \end{array}$$

If $|\epsilon_{1}|>\Phi_{1}/k_{1}$, then ${\dot{V}}_{1}\leq 0$. Therefore, we can conclude that $|\epsilon_{1}|\leq {\bar{\epsilon }}_{1}$ where ${\bar{\epsilon }}_{1}=\max\left\{\epsilon_{1}\left(0\right),\Phi_{1}/k_{1}\right\}$, and $z_{1}$ satisfies $|z_{1}|<1$. Furthermore, the boundedness of $\epsilon_{1}$ and *μ* implies that *α* is bounded, and thus, ${\dot{\epsilon }}_{1}$ and $\dot{\mu }$ are bounded. From Equations (6) and (7), $\dot{\alpha }$ is also bounded.

*Step 2*: Consider the following Lyapunov function candidate for $\epsilon_{2}$.

(14) $$\begin{array}{c} V_{2}=\frac{1}{2}\epsilon_{2}^{2} \end{array}$$

The time derivative of Equation (14) along with Equation (7) is

(15) $$\begin{array}{c} {\dot{V}}_{2}=\frac{\delta_{2}}{\rho_{2}}\epsilon_{2}\left\{f\left(x_{1},x_{2}\right)+b\left(x_{1}\right)\tau -\dot{\alpha }+k_{\mu }\mu -\gamma_{1}\tanh\left({\dot{\theta }}_{w}\right)-\tanh\left(\epsilon_{2}/2\right){\dot{\rho }}_{2}\right\}\\ \;\;\;\;=\frac{\delta_{2}}{\rho_{2}}\epsilon_{2}\left\{f\left(\tanh\left(\epsilon_{1}/2\right)\rho_{1},\tanh\left(\epsilon_{2}/2\right)\rho_{2}\right)+b\left(x_{1}\right)\tau -\dot{\alpha }+k_{\mu }\mu -\tanh\left({\dot{\theta }}_{w}\right)-\tanh\left(\epsilon_{2}/2\right){\dot{\rho }}_{2}\right\} \end{array}$$

where $\delta_{2}=2\cosh^{2}\left(\epsilon_{2}/2\right)>0$. The actual control law *τ* is chosen as

(16) $$\begin{array}{c} \tau =N(\zeta )\eta \\ \eta =k_{2}\epsilon_{2}+\gamma_{2}\frac{\delta_{2}\epsilon_{2}}{2\rho_{2}}+\frac{k_{\mu }\mu \rho_{2}}{\delta_{2}}\\ \dot{\zeta }=\frac{\delta_{2}}{\rho_{2}}\eta \epsilon_{2} \end{array}$$

where $k_{2}$ and $\gamma_{2}$ are positive constants.

**Remark 4.**  In Equation (16), the actual control law does not require any function approximations to compensate the uncertainties. Further, the differentiation of the virtual control is not required in spite of using the backstepping technique. Therefore, the controller is simple compared with previous results for the Segway.

Substituting Equation (16) into Equation (15) yields

(17) $${\dot{V}}_{2}=\frac{\delta_{2}}{\rho_{2}}\epsilon_{2}\left\{b\left(x_{1}\right)N\left(\zeta \right)\eta +f\left(\tanh\left(\epsilon_{1}/2\right)\rho_{1},\tanh\left(\epsilon_{2}/2\right)\rho_{2}\right)-\dot{\alpha }+k_{\mu }\mu -\gamma_{1}\tanh\left({\dot{\theta }}_{w}\right)-\tanh\left(\epsilon_{2}/2\right){\dot{\rho }}_{2}\right\}$$

In Step 1, the boundedness of $\epsilon_{1}$ and $\dot{\alpha }$ is proved. Since f(·) is composed of $\tanh\left(\epsilon_{1}/2\right)\rho_{1}$ and $\tanh\left(\epsilon_{2}/2\right)\rho_{2}$, it is bounded. Then, there exists a positive constant $\Phi_{2}$ satisfying $|f-\dot{\alpha }-\gamma_{1}\tanh\left({\dot{\theta }}_{w}\right)-\tanh\left(\epsilon_{2}/2\right){\dot{\rho }}_{2}|\leq \Phi_{2}$. Thus Equation (17) can be expressed as

(18) $$\begin{array}{c} {\dot{V}}_{2}\leq \frac{\delta_{2}}{\rho_{2}}(b\left(x_{1}\right)N\left(\zeta \right)\eta \epsilon_{2}+k_{\mu }\mu +\Phi_{2}|\epsilon_{2}\left|\right)=b\left(x_{1}\right)N\left(\zeta \right)\dot{\zeta }+\frac{\delta_{2}}{\rho_{2}}(k_{\mu }\mu +\Phi_{2}|\epsilon_{2}\left|\right) \end{array}$$

Note that $\dot{\zeta }=\frac{\delta_{2}}{\rho_{2}}\eta \epsilon_{2}=\frac{\delta_{2}}{\rho_{2}}\epsilon_{2}\left(k_{2}\epsilon_{2}+\frac{\gamma_{2}\delta_{2}\epsilon_{2}}{2\rho_{2}}+\frac{k_{\mu }\mu \rho_{2}}{\delta_{2}}\right)$. Adding and subtracting $\dot{\zeta }$ in the right side of Equation (18), we have

(19) $$\begin{array}{c} {\dot{V}}_{2}\leq b\left(x_{1}\right)N\left(\zeta \right)\dot{\zeta }+\dot{\zeta }-\frac{\delta_{2}}{\rho_{2}}k_{2}\epsilon_{2}^{2}-\frac{\gamma_{2}\delta_{2}^{2}\epsilon_{2}^{2}}{2\rho_{2}^{2}}+\frac{\delta_{2}}{\rho_{2}}\Phi_{2}\left|\epsilon_{2}\right| \end{array}$$

By the inequality,

$$\begin{array}{c} -\frac{\gamma_{2}\delta_{2}^{2}\epsilon_{2}^{2}}{2\rho_{2}^{2}}+\frac{\delta_{2}}{\rho_{2}}\Phi_{2}\left|\epsilon_{2}\right|\leq \frac{\Phi_{2}^{2}}{2\gamma_{2}} \end{array}$$

Then, Equation (19) can be rewritten as

(20) $$\begin{array}{c} {\dot{V}}_{2}\leq -c_{0}V_{2}+b\left(x_{1}\right)N\left(\zeta \right)\dot{\zeta }+\dot{\zeta }+c_{1} \end{array}$$

where $c_{0}=\frac{2k_{2}}{\rho_{2}\left(0\right)}$ and $c_{1}=\frac{\Phi_{2}^{2}}{2\gamma_{2}}$. Multiplying $e_{0}^{c}t$ on both sides of Equation (20) yields,

(21) $$\begin{array}{c} \frac{d}{dt}\left(V_{2}e^{c_{0}t}\right)\leq \left(bN\left(\zeta \right)\dot{\zeta }+\dot{\zeta }+c_{1}\right)e^{c_{0}t} \end{array}$$

Integrating Equation (21) on [0,t], we have

(22) $$\begin{array}{c} V_{2}\left(t\right)\leq V_{2}\left(0\right)e^{-c_{0}t}+\int_{0}^{t}\left\{bN\left(\zeta \right)+1\right\}\dot{\zeta }e^{-c_{0}\left(t-\varrho \right)}d\varrho +\int_{0}^{t}c_{1}e^{-c_{0}\left(t-\varrho \right)}d\varrho \\ \;\;\;\;\;\;\;\;\;\leq c_{2}+e^{-c_{0}t}\int_{0}^{t}bN\left(\zeta \right)\dot{\zeta }e^{c_{0}\varrho }d\varrho +e^{-c_{0}t}\int_{0}^{t}\dot{\zeta }e^{c_{0}\varrho }d\varrho \end{array}$$

where $c_{2}=V_{2}\left(0\right)+\frac{c_{1}}{c_{0}}$. Note that $c_{1}$ and $c_{2}$ are positive. By Lemma 1, we can conclude that $V_{2}\left(t\right)$, *ζ* and $\epsilon_{2}$ are bounded on $\left[0,t_{f}\right)$. The boundedness of $\epsilon_{2}$ implies that $z_{2}$ satisfies $|z_{2}|<1$. According to [28], the boundedness of these signals ensures $t_{f}=\infty$.

**Theorem 1.**  For the Segway Equation (3) with completely unknown time-varying control coefficient and model uncertainties, if we apply the controller Equation (16), then the solution of the closed-loop system is bounded. Furthermore, the errors remain within their prescribed performance functions such that $|x_{1}|<\rho_{1}$ and $|x_{2}-\alpha -\mu |<\rho_{2}$.

**Proof of Theorem 1.**  By the previous design procedures from Step 1 to Step 2, it is proved that $\epsilon_{1}$ and $\epsilon_{2}$ are bounded. Thus, $|z_{1}|<1$ and $z_{2}<1$. This means that $|x_{1}|<\rho_{1}$ and $|x_{2}-\alpha -\mu |<\rho_{2}$. ☐

It is necessary to prove the convergence of ${\dot{\theta }}_{w}$. For the simplicity, assume that $\epsilon_{1}$ and $\epsilon_{2}$ converge to zero. Since the bounds of $\epsilon_{1}$ and $\epsilon_{2}$ are depend on $k_{1}$ and $k_{2}$, the bounds of them can converge to nearby zero if we increase $k_{1}$ and $k_{2}$. The convergence of $\epsilon_{1}$ and $\epsilon_{2}$ leads to the convergence of $z_{1}$ and $z_{2}$. From Equations (4) and (11), $x_{1}$ and $x_{2}$ also converge to zero. This implies that ${\dot{x}}_{1}$ and ${\dot{x}}_{2}$ are zero, and thus, control torque *τ* is zero from Equation (3). Then, from Equation (16), *η* is zero because *ζ* is bounded due to $\dot{\zeta }=0$. Since *η* is composed of $\epsilon_{2}$ and *μ* in Equation (16), *μ* converge to zero. If *μ* is bounded and converges to zero as t→∞, the angular velocity $\theta_{w}$ of the wheel converges to zero by Equation (6) and Lemma 2 presented in [7].

**Remark 5.**  *The design procedure is as follows: (i) select $\rho_{1}\left(0\right)$ to satisfy the condition such that $\rho_{1}\left(0\right)>\left|x_{1}\left(0\right)\right|$; (ii) select $l_{1}$ and $\rho_{1}\left(\infty \right)$ to satisfy the convergence rate and robustness for the external disturbance after it is stabilized, respectively; (iii) calculate $z_{1}\left(0\right)$ using Equation (4); (iv) select $k_{1}$ properly. The error $\epsilon_{1}$ will be decreased as $k_{1}$ is increased. Calculate the virtual control α using Equation (11); (v) select $\rho_{2}\left(0\right)$ to satisfy the condition such that $\rho_{2}\left(0\right)>\left|x_{2}\left(0\right)-\alpha \left(0\right)-\mu \left(0\right)\right|$; (vi) select $l_{2}$ and $\rho_{2}\left(\infty \right)$ to satisfy the convergence rate and robustness for the external disturbance, respectively; (vii) calculate $z_{2}\left(0\right)$ using Equation (4); (viii) select $k_{2}$ properly. Increasing $k_{2}$ leads to the smaller error $\epsilon_{2}$. Calculate the actual control τ using Equation (16).*

## 4. Simulation Results

In this section, the simulation results are provided to illustrate the effectiveness of the proposed scheme. For the real application, we use the model parameters presented in [29]. These are only for the simulation. That is, the proposed control scheme does not require the exact information of model parameters for the application and the simulation results show the robustness against these model uncertainties. The control parameters are chosen as $l_{1}=l_{2}=1$, $\rho_{1}\left(0\right)=\rho_{2}\left(0\right)=10$, $\rho_{1}\left(\infty \right)=\rho_{2}\left(\infty \right)=2.5$, $k_{1}=10$, $k_{2}=500$, $k_{\mu }=15$, $\gamma_{1}=35$, and $\gamma_{2}=1$.

**Figure 2:**
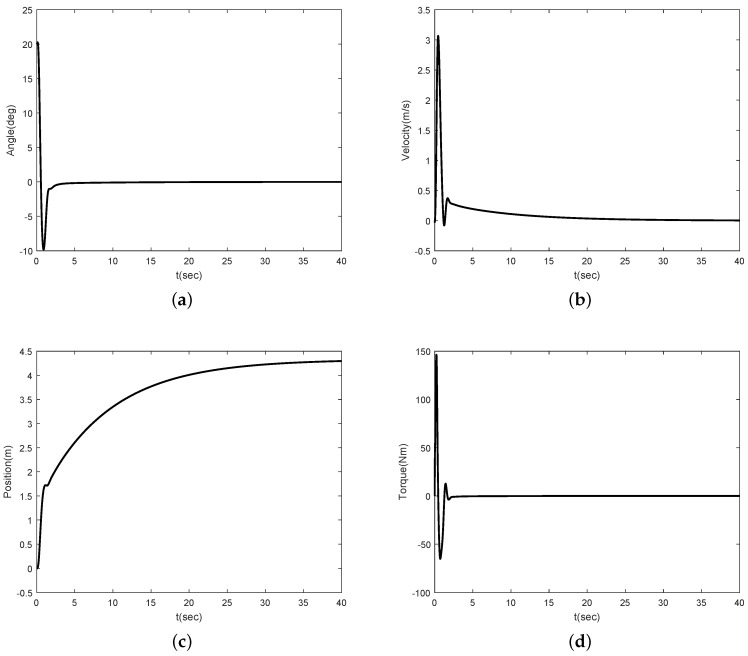
Simulation result for $\theta \left(0\right)=20^{\circ }$: (**a**) angle *θ*; (**b**) linear velocity *v*; (**c**) position *x*; (**d**) torque *τ*.

**Figure 3:**
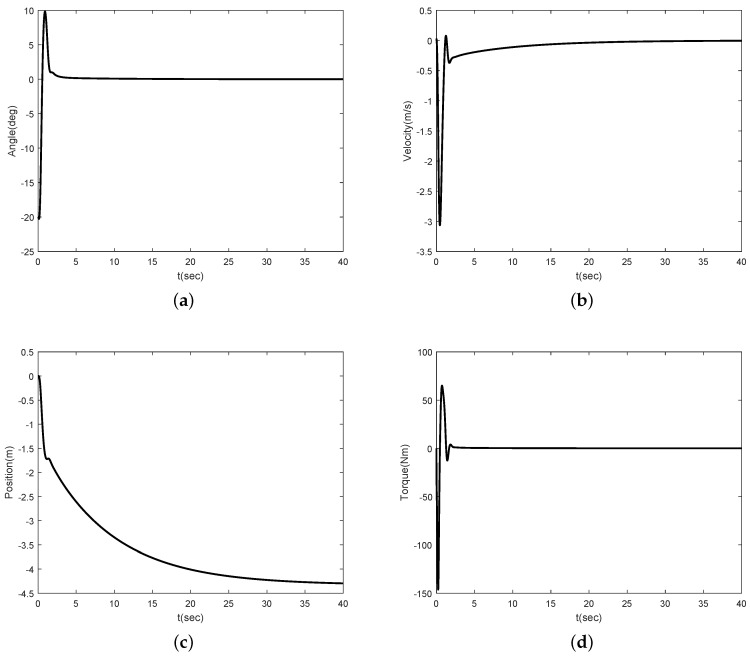
Simulation result for $\theta \left(0\right)=-20^{\circ }$: (**a**) angle *θ*; (**b**) linear velocity *v*; (**c**) position *x*; (**d**) torque *τ*.

**Figure 4:**
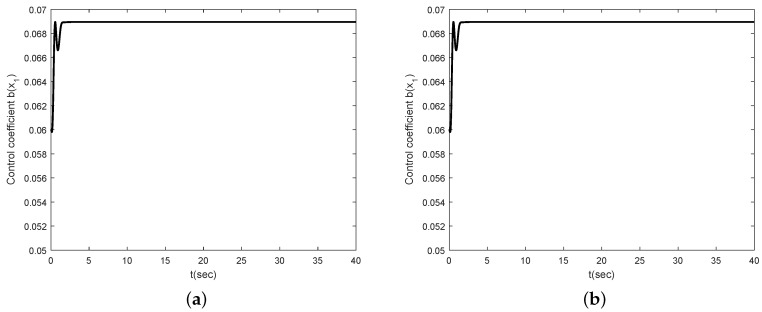
Control coefficient $b(x_{1})$: (**a**) $\theta \left(0\right)=20^{\circ }$; (**b**) $\theta \left(0\right)=-20^{\circ }$.

**Figure 5:**
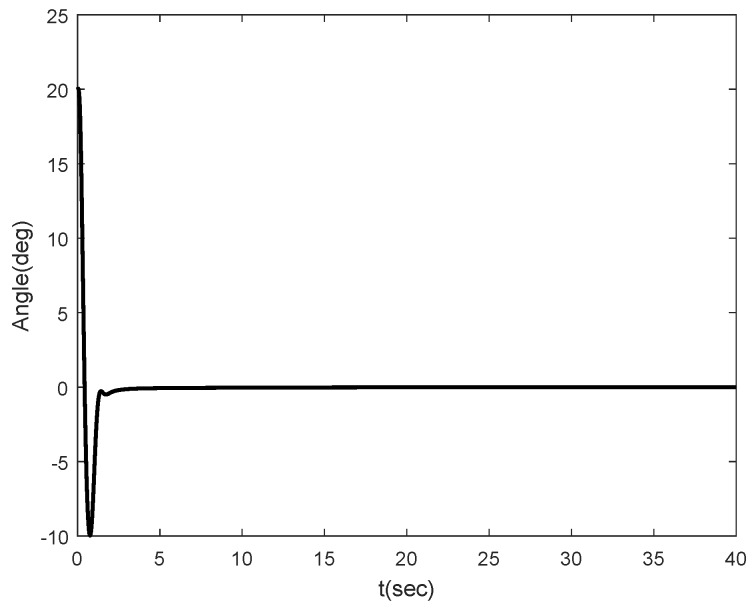
Angle of segway, m = 40 kg, l = 0.75 m.

Simulation results are shown in Figure 2, Figure 3, Figure 4 and Figure 5. Figure 2 and Figure 3 show the simulation results for $\theta \left(0\right)=20^{\circ }$ and $\theta \left(0\right)=-20^{\circ }$, respectively. Figure 2a,b show that the angle of the inclination and control torque converge to zero as times go on. This means that the proposed control scheme is well working for the Segway model. Figure 2b,c show the position and velocity of the Segway, respectively. As one can see, the velocity of the Segway converges to zero because the angle of the inclination is zero. Thus, we can know that the Segway does not move if the control objective, which should return to the vertical after the initial disturbance, is achieved. Figure 3 also show that the angle of the inclination converges to zero in the case of the opposite direction. Figure 4 depicts the control coefficient $b(x_{1})$ for both two cases. The control coefficients are time-varying while the angle of the inclination is not zero. On the other hand, these become constants because *θ* is time-invariant after the convergence. To show the effectiveness of the proposed control scheme even though a rider is changed, we simulate other model parameters such as m=40 kg and l=0.75 m. Figure 5 shows the simulation result. Compared with Figure 2a, there is no different in the performance between them.

**Figure 6:**
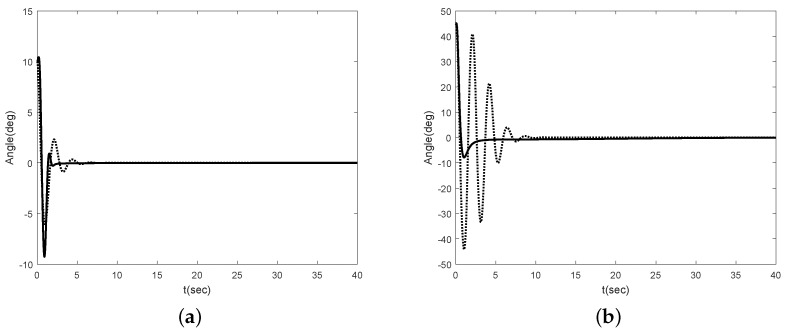
Angle of Segway without disturbance (solid : proposed method, dotted : LQR method): (**a**) $\theta \left(0\right)=10^{\circ }$; (**b**) $\theta \left(0\right)=45^{\circ }$.

**Figure 7:**
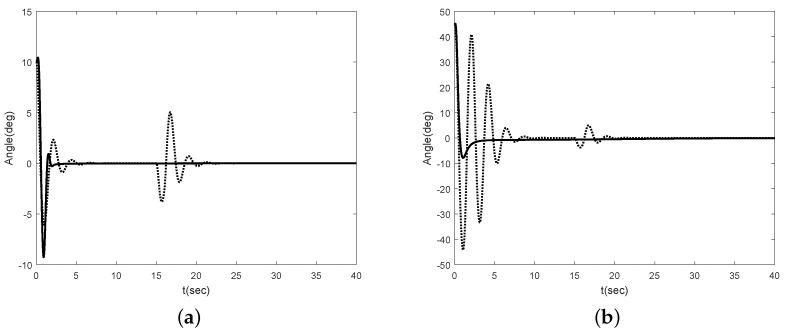
Angle of Segway with disturbance (solid : proposed method, dotted : LQR method): (**a**) $\theta \left(0\right)=10^{\circ }$; (**b**) $\theta \left(0\right)=45^{\circ }$.

To compared with previous results, we simulate using LQR method presented in [22] under the same model parameters. The simulation results are shown in Figure 6 and Figure 7. Figure 6 shows the angle of the Segway without disturbance for $\theta \left(0\right)=10^{\circ }$ and $\theta \left(0\right)=45^{\circ }$. In [22], they use the linearized model, i.e., the Segway model is linearized at $\theta \left(0\right)=0^{\circ }$. Thus, there is no difference in the performance at $\theta \left(0\right)=10^{\circ }$. However, if the initial error is large enough, we can see that there is a performance difference between our method and [22]. Figure 6b shows this result. Figure 7 shows the angle of the Segway with disturbance. To show the robustness of the proposed scheme after it is stabilized, we apply the external disturbance to the Segway from time 15 to 16 s. As one can see, the proposed scheme is effective even though the external disturbance is applied to the Segway after it is stabilized. Therefore, we can conclude that the proposed scheme has the good performance even though there are unknown control coefficient and model uncertainties.

## 5. Conclusions

In this paper, a robust controller has been proposed for the Segway with unknown time-varying control coefficient and model uncertainties. To deal with unknown time-varying control coefficient and model uncertainties, we design the controller using the Nussbaum technique and prescribed performance function. Since the proposed control scheme does not require the adaptive technique, neural network, and fuzzy logic to compensate the uncertainties, the structure of the controller is simple. Furthermore, to solve the underactuated problem, we introduce the auxiliary variable that is used to control the velocity of the Segway. From the Lyapunov stability theory, we prove that all error signals of the closed-loop control system are bounded. Finally, the simulation results show that the proposed scheme has better performance compared with previous results.
